# A Validated HPTLC Method for Determination of Ondansetron in Combination with Omeprazole or Rabeprazole in Solid Dosage Form

**DOI:** 10.4103/0250-474X.43011

**Published:** 2008

**Authors:** P. B. Raval, Manisha Puranik, S. J. Wadher, P. G. Yeole

**Affiliations:** P.G. Department of Quality Assurance, Institute of Pharmaceutical Education and Research, Borgaon (Meghe), Wardha-442 001, India

**Keywords:** Ondansetron, rabeprazole, omeprazole, HPTLC, validation

## Abstract

A simple, precise, accurate and rapid high performance thin layer chromatographic method has been developed for the simultaneous estimation of ondansetron combinations in solid dosage form with omeprazole and rabeprazole, respectively. The method involved separation of components by TLC on a precoated silica gel 60 F_254_ using a mixture of dichloromethane:methanol (9:1) as a mobile phase. Detection of spots was carried out at 309 nm and 294 nm for ondansetron with omeprazole and ondansetron with rabeprazole combinations, respectively. The mean retardation factor for ondansetron and omeprazole were found to be 0.42±0.02, 0.54±0.03, respectively while for ondansetron and rabeprazole, 0.41± 0.02 and 0.51±0.02, respectively. The linearity and range was 0.1 to 0.5 μg/spot for three drugs. The method was validated for precision, accuracy and reproducibility.

Ondansetron combination with proton pump inhibitors has recently been introduced in market for the treatment of peptic ulcer, gastroesophagal reflux disease (GERD) and to prevent nausea. Ondansetron antagonizes 5HT-3 receptor both peripherally as well as centrally and block the initiation of the reflux, so is used as antiematic. Ondansetron is official in USP^1^. Omeprazole and rabeprazole are benzimidazole proton pump inhibitors, which suppress gastric acid secretion by H^+^/K^+^ -ATPase enzyme system at the secretory surface of the gastric parietal cell. These drugs are used for the treatment of duodenal, gastric and esophageal ulceration. Omeprazole is official in USP^1^, IP^2^, BP^3^, where as rabeprazole is not official in any of the pharmacopoeia, but is reported in Merck Index^4^.

Literature survey revealed HPLC^5^,^6^ in human plasma, visible spectrophotometric method^7^ for ondansetron in solid dosage form. For omeprazole methods reported are HPLC-MS and HPLC-UV in biological fluids^8^,^9^, capillary electrophoresis^10^, HPLC employing electrochemical and coulometric detection^11^, TLC^12^ and spectrophotometry^13^. For rabeprazole method reported are HPLC for detection in blood plasma^14^,^15^ spectrophotometric methods^16^, and LC-MS/MS^17^ method. As no analytical method has so far been indicated for the ondansetron combinations with proton pump inhibitors, an attempt has been made to estimate them simultaneously by HPTLC.

Ondansetron reference standard was obtained as a gift sample from Neon Lab Ltd, Mumbai. Rabeprazole was obtained from Dr. TTTTy’s Lab, Hyderabad and omeprazole obtained from Zydus Cadila Health Care, Ahmedabad. Silica gel 60 F_254_ TLC plates (20 × 10cm, layer thickness 0.2 mm, E. Merk, Mumbai) were used as a stationary phase. Dichloromethane:methanol (9:1) used as a mobile phase for both combinations. A Camag HPTLC system comprising of Camag linomat V semiautomatic sample applicator, Camag TLC scanner 3, Camag Win CATS software, Camag twin trough chamber and a sonicator were used. Tablets containing omeprazole (10 mg) and ondansetron (4 mg) (Ranidom-O, Mankind Pharmaceuticals Pvt. Ltd., Mumbai, India) and capsules containing rabeprazole (20 mg) and ondansetron (6 mg) (OND-R, Besto Chemical Ltd., New Delhi, India) were used during study.

Working standards of ondansetron, omeprazole and rabeprazole (25 mg each) were weighed and diluted with methanol to get the final concentration 0.05 μg/μl for omeprazole and 0.02 μg/μl for ondansetron and 0.05 μg/μl for rabeprazole and 0.015 μg/μl for ondansetron. For ondansetron combination with rabeprazole, contents of twenty capsules was crushed to fine powder, quantity equivalent to 20 mg rabeprazole (6 mg ondansetron) was weighed accurately and transfer to 10 ml volumetric flask and for ondansetron combination with omeprazole, twenty tablets were crushed to fine powder, weight equivalent to 10 mg of omeprazole (4 mg of ondansetron) was transferred to 10 ml volumetric flask. Then to each flask about 5ml of methanol was added and sonicated for 15 min, finally volume was made to mark with methanol. The extracts were filtered through Whatman filter paper 41 and required dilutions were made to get the final concentration containing 0.05μg/μl rabeprazole, 0.015 μg/μl ondansetron and 0.05 μg/μl omeprazole, 0.02 μg/μl ondansetron and 6 μl of standard and sample were applied as 5 mm band on the TLC plate.

TLC plates were prewashed with methanol and activated prior to use. The chromatographic conditions maintained were: Precoated Silica gel 60 F_254_ (20×10 cm) aluminum sheets as stationary phase. Dichloromethane:methanol (9:1 V/V) as mobile phase for both the ondansetron combinations. Samples were applied as bands 5 mm width at 14.1 mm intervals using Camag linomate V semiautomatic sample applicator and migration distance allowed was 80 mm, drying of plate done for 3 min at 60^°^ temperatures. The plates were scanned at 309 nm for omeprazole and ondansetron and 294 nm for rabeprazole and ondansetron combination with Camag TLC scanner III, using Camag Win CATS software (fig. 1).

**Fig. 1:**
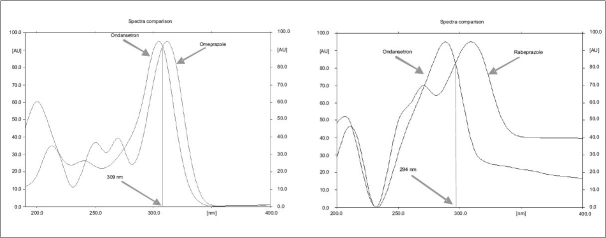
UV spectra of ondansetron in combinations UV spectra of ondansetron in combinations with (a) omeprazole and (b) rabeprazole, respectively

For calibration curve, 0.1, 0.2, 0.3, 0.4 and 0.5 μg/μl standard solution of omeprazole, rabeprazole and ondansetron were applied on TLC plate. The TLC plates were dried, developed and analyzed as described earlier.

Filtered solutions (6 μl) of the marketed formulations were spotted on to the plate followed by development scanning. The analysis was repeated six times, the spot was resolved into two peaks in the chromatogram of drug samples. The contents were calculated from the peak areas of standards and samples recorded.

A solvent system that would give dense and compact spots with appropriate and significantly different R_f_ values was desired for quantification of ondansetron combinations. The mobile phase consisting of dichloromethane: methanol (9:1 V/V) gave R_f_ value of 0.42±0.02, 0.54±0.03, respectively for ondansetron and omeprazole while for ondansetron and rabeprazole, 0.41± 0.02 and 0.51±0.02, respectively (fig. 2).

**Fig. 2:**
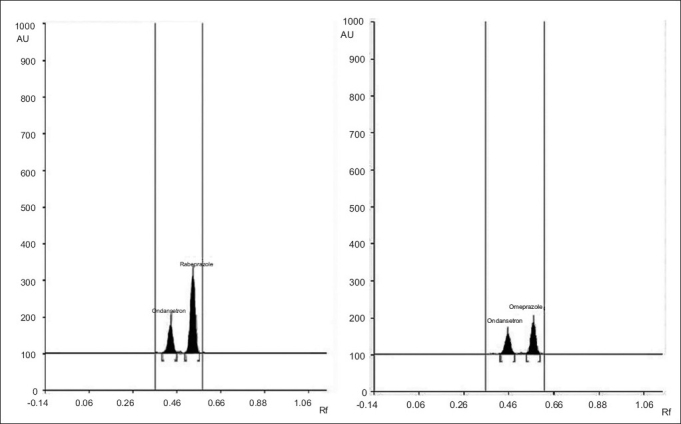
A typical HPTLC chromatogram of ondansetron combinations A typical HPTLC chromatogram of ondansetron combinations with (a) rabeprazole and (b) omeprazole, respectively in the marketed formulations

The developed method was validated in terms of linearity and range, limit of detection, limit of quantification, recovery study, inter days study, intra day study and study by different analysts. The limit of detection for omeprazole, ondansetron and rabeprazole, ondansetron was found to be 99 ng/spot, 39.9 ng/spot, and 90.3 ng/spot, 54.2 ng/spot, respectively. The limit of quantification for omeprazole, ondansetron and rabeprazole, ondansetron was found to be 302.8 ng/spot, 121.1 ng/spot and 273.9 ng/spot, 164.4 ng/spot, respectively.

The linear regression data (n=6, Table 1) showed a good linear relationship over a concentration range 100 to 500 ng/spot for omeprazole, ondansetron and rabeprazole. Repeatability of method was determined by 6 times spotting 10 μl of standard drug solution on TLC plate, measurement of peak areas was performed and from the peak areas the % RSD was determined. For omeprazole and ondansetron % RSD was found to be 0.35 and 0.40, respectively and for rabeprazole and ondansetron % RSD was 0.19 and 0.45, respectively. Repeatability of measurement was determined by spotting 10μl of standard drug solution on TLC plate, after development the separated spots were scanned six times without changing position and % RSD for measurement of peak areas of omeprazole and ondansetron was found to be 0.64 and 0.80, respectively and for rabeprazole and ondansetron % RSD was 0.31 and 0.42, respectively.

**TABLE 1:** VALIDATION PARAMETERS

Parameters*	Omeprazole	Ondansetron	Rabeprazole	Ondansetron
Rf (±SD)	0.54±0.03	0.42±0.02	0.51±0.02	0.41±0.02
Linearity and range (ng/spot)	100-500	100-500	100-500	100-500
Limit of detection (ng/spot)	99	39.9	90.3	54.2
Limit of quantification (ng/spot)	302.8	121.1	273.9	164.4
Repeatability of application (%RSD)	0.35	0.40	0.19	0.45
Repeatability of measurement (%RSD)	0.64	0.80	0.31	0.42
Intra day (%RSD)	0.12	0.14	0.66	0.29
Inter day (%RSD)	0.77	0.58	0.23	0.54
Different analysts (%RSD)	0.21	0.61	0.14	0.72

* Average of 6 determinations

The assay value for the marketed formulation was found to be within the limits as listed in Table 2. The low RSD value indicates suitability of the method for routine analysis of omeprazole, ondansetron and rabeprazole, ondansetron in pharmaceutical dosage form. Recovery studies were carried out to study accuracy and precision of the method. These studies were carried out at three levels i.e. multiple level recovery studies. To the powder formulations the pure standard drug were added at 80%, 100% and 120% levels, dilutions were made and analyzed by the method, the % recovery was calculated by using formula, % recovery = (T-A)/S × 100 where, T is total amount of the drug estimated, A is the amount of drug contributed by tablet powder and S is the amount of pure drug added. The results of recovery studies for both the ondansetron combinations were found to be around 99-100%, indicating that the method is free from interference from excipients.

**TABLE 2:** ANALYSIS OF ONDENSETRON WITH OMEPRAZOLE (RANIDOM-O) AND ONDENSETRON WITH RABEPRAZOLE (OND-R)

Brand name	Drug	Label claim (mg/tab)	Amount found* (mg/tab)	% drug found*	% RSD
Ranidom-O	Ondensetron	4	3.99	99.90	0.45
	Omeprazole	10	10.039	100.39	0.49
OND-R	Ondensetron	6	5.99	99.96	0.82
	Rabeprazole	20	19.96	99.83	0.72

* Average value of 6 determinations

The ruggedness of the method was evaluated by studying analyst to analyst, intra day and inter days variations and the % RSD was calculated, that was found to be within range. From the above results it can be concluded that the HPTLC method is accurate, precise, specific and reproducible and can be used for routine analysis of ondansetron combinations with proton pump inhibitors in solid dosage form.
